# Development and initial validation of the Northwick Park Therapy Dependency Assessment

**DOI:** 10.1177/0269215509337447

**Published:** 2010-03

**Authors:** Lynne Turner-Stokes, Asa Shaw, Janet Law, Hilary Rose

**Affiliations:** King's College London, School of Medicine, Department of Palliative Care, Policy and Rehabilitation and Regional Rehabilitation Unit, Northwick Park Hospital; Regional Rehabilitation Unit, Northwick Park Hospital, Harrow, UK

## Abstract

**Objectives**: To describe the development and initial validation of the Northwick Park Therapy Dependency Assessment (NPTDA) as a measure of therapy interventions in neurorehabilitation.

**Design**: An iterative development process, followed by comparison with systemic prospective activity analysis, and parallel application of prospective and retrospective scores

**Setting**: A tertiary specialist inpatient neurorehabilitation service

**Participants**: A total of 37 patients (M:F 21:16, mean age 41.8 (SD 14.7) years) with complex neurological disability in two consecutive cross-sectional cohorts.

**Methods**: The NPTDA was developed and refined over 18 months, together with an algorithm that converts ordinal scores to estimated therapy hours/week. NPTDA-estimated hours were compared with ‘actual' therapy hours/week, identified from activity analysis. In a subsequent cohort analysis, prospectively rated NPTDA scores (reflecting intended levels of intervention) were compared with retrospective NPTDA scores (actual interventions).

**Results**: NPTDA-estimated therapy hours/week were strongly correlated with those identified from activity analysis, for total scores (Spearman rho 0.77, *P* < 0.0001), and also for all five subdomains for direct (hands-on) intervention (rho 0.70–0.93, *P* < 0.0001). The initial test algorithm overestimated therapy hours (Wilcoxon *z* = ⊟3.9, *P* < 0.001). After adjustment, reanalysis using a revised algorithm showed this bias to be removed (Wilcoxon *z* = ⊟1.4 *P* = 0.15). Prospective and retrospectively applied total NPTDA scores were strongly correlated (rho 0.61, *P* < 0.0001). Although intended levels of intervention were higher than those actually delivered (Wilcoxon *z* = ⊟3.30, *P* < 0.001), the differences corresponded to real deviations from intended practice.

**Conclusion**: In this initial evaluation, after revision of the algorithm, the NPTDA provided acceptable estimate of therapy interventions. Further evaluation is now required in other populations and settings.

## Introduction

A substantial literature now supports the benefits of higher intensity rehabilitation, at least for certain patients,^1–3^ but ‘higher intensity' has yet to be properly defined. Patients with neurological disabilities have widely varying needs for rehabilitation, often involving several disciplines. Simply recording hours of therapy input has little meaning unless the nature of interventions can be also be described. Many authors have called for practice-based research to ‘open the black box', in order to provide clearer description of the rehabilitation content.^4^ A number of tools have been developed to facilitate the systematic recording of therapy interventions,^5–12^ which include tools to describe the type of interventions offered for patients with stroke^5,7–10^ and spinal cord injury.^11,12^ However, these can only be applied to describe interventions that were *actually given*, rather than looking at what might be *needed*. Moreover, existing tools focus only on physical interventions (physiotherapy, occupational therapy and in some cases speech and language therapy^8^) and omit other interventions such as psychology, dietetics and social work, which play an important role in holistic neurological rehabilitation programmes.

Since the mid-1990s, work has been underway at Northwick Park Hospital in the UK to develop a comprehensive set of tools for rehabilitation, which are practical to apply in the course of routine clinical practice; and which may be used to measure nursing and therapy intervention, in relation to need, and to quantify this in terms of staff time. A common underlying principle of these instruments is that they are designed to be applied both prospectively to measure ‘needs' for rehabilitation intervention, and retrospectively to describe what the patient actually ‘gets', so that in future they could be applied as a framework for quantifying gaps in service provision. They also include a computerized algorithm, which translates the description of dependency into a generic estimation of implications for staff time.

The Northwick Park Nursing Dependency Scale (NPDS) was published in 1999 as a tool to assess nursing needs of patients in rehabilitation settings. It translates, by means of a computerized algorithm (the Northwick Park Care Needs Assessment) into an estimate of care hours required.^13^ It is shown to be a valid measure of nursing needs^14,15^ and has been increasingly applied in the context of routine clinical practice in the UK,^16^ as well as abroad.^17^

In 2004, a project grant was awarded by the UK Department of Health (Grant ref. 030/0066) to develop an equivalent tool to assess therapy dependency.^18^ The Northwick Park Therapy Dependency Assessment (NPTDA) was developed through an iterative process over two years. This paper provides a brief description of its development and initial validation.

## Methods

### Tool development

The setting for this development and initial evaluation was the Regional Rehabilitation Unit at Northwick Park Hospital. The unit provides a tertiary specialist inpatient neurorehabilitation service for younger adults (mainly aged 16–65 years) with complex neurological disabilities. An experienced multidisciplinary staff team includes specialty-trained rehabilitation doctors and nurses; and a range of allied health professions which include physiotherapy, occupational therapy, speech and language therapy, psychology, dietetics, and social work, all of whom contributed to the project. Ethics permission was obtained from the Local Research Ethics Committee.

Development of the NPTDA involved an iterative process of consultation with senior multidisciplinary team members to identify the factors that describe requirements for different levels of therapy intervention. This led to the development of a draft tool in 2004. Over the next 18 months, successive periods of cross-sectional application of the tool provided an extensive prospective observational dataset. Refinement through serial analysis and team reflection in the context of clinical use ensured content validity for this setting, and the NPTDA evolved to its final form in late 2005. A full description of the early development and testing process is beyond the scope of this article, but is detailed in the Department of Health project report.^18^

### The Northwick Park Therapy Dependency Assessment (NPTDA) tool

The NPTDA is a measure of therapy intervention designed for use in specialist neuro-rehabilitation settings, where high intensity rehabilitation is provided by a multidisciplinary team.

Key principles of the tool are as follows:

- It includes 30 items of therapy dependency in seven domains (A–G), which are shown in Appendix 1. The total range of the score is 0–100.
  – Items in domains A–E record *direct* ‘hands-on' patient care. They are each scored on a range of 0–4, according to the general scale structure illustrated in Appendix 1.
  –Items in domain F record *indirect* patient-related care (e.g. attending meetings, writing reports, etc. which may be conducted away from the patients), and additional activities such as groups or staff-escorted clinic attendance. These items are scored on a range of 0–2.
  –Items in domain G are ‘text only' and record the use of special facilities/equipment, investigations and procedures, for the purpose of audit and coding.
- Each patient is rated individually, the scores for each item being based on the interventions for a one-week period. A scoring manual provides detailed level descriptions for each item. Therapists are encouraged to rely primarily on these descriptions, but in order to provide a rough guide to assist scoring, approximate time ranges have also been ascribed to each scoring level (see Appendix 1b). These were defined through observational analysis during development and they vary somewhat across the different items.
- The data are entered into an electronic database which applies a computerized algorithm to estimate the therapy hours for each level of each item. (This paper will describe how the algorithm was developed.)
  –For score levels 1–3, the algorithm applies predetermined hours which are allocated to the lead discipline identified. A default lead discipline is suggested for each item, but this may be changed to reflect normal practice within a given setting.
  –Level 4 (and level 3.5, which was added as a result of this evaluation) reflect interdisciplinary working where several different disciplines are working in collaboration on the same task area (item). In this case, the hours are specified individually for each discipline on the scoring sheet at the time of rating.
- The allocated times are summed to provide an estimate of the total therapy hours and also provide a breakdown of hours for each discipline.

As noted above, the NPTDA is designed to be applied in various ways depending on the intended purpose of measurement. For the assessment of *therapy needs*, NPTDA scores may be applied prospectively, based on the judgement of the therapy team in respect of the level of input required. For the assessment of *therapy interventions*, NPTDA scores may be applied retrospectively, based on the levels of intervention actually provided. In this way it is theoretically possible to record both, and to compare the needs for intervention with the levels of input provided (see Discussion).

### Validation

This initial validation took part in two stages:

1. In the first stage, we validated the NPTDA scores, and refined the conversion algorithm for translating raw scores into therapy hours, by comparing retrospectively applied NPTDA estimates of therapy intervention with the actual hours of therapy intervention – recorded through parallel systematic activity analysis.
2. In the second stage, using a subsequent cohort of patients, we compared prospective and retrospective NPTDA ratings, recorded in parallel for the same treatment period, to examine the validity of prospective application.

### Stage 1: Comparison of NPTDA-estimated hours with activity analysis

#### Design and participants

In a cross-sectional cohort analysis, routinely rated NPTDA scores for all inpatients on the unit were compared with the results of activity analysis for the same period (four consecutive working weeks between 21 November 2005 and 16 December 2005). All 24 therapists (20.3 whole-time equivalents) working on the unit at the time participated. Disciplines included physiotherapy, occupational therapy, speech and language therapy, dietetics, psychology and social work. The patient cohort consisted of 8 women and 9 men: mean age 45.5 years (SD 17.1). All had complex neurological disabilities arising from acquired brain injury (8 strokes, 5 traumatic), spinal cord injury (*n* = 2) or Guillain-Barré syndrome (*n* = 2).

#### Data collection

Data were collected in the course of routine clinical practice. Patients on the unit are normally divided into two teams (‘Red' and Blue') and the weekly ward round alternates between the teams, so that each patient is reviewed fortnightly. Inevitably there were admissions and discharges during the four-week study period, so that 12 patients were rated on two occasions and 5 were rated only once, giving a total of 29 parallel sets of ratings of NPTDA scores with activity analysis for the corresponding period.

NPTDA scores were applied during the weekly ward round, by the treating team. They were rated retrospectively for each patient to reflect a week's therapy intervention, based on the average of the previous two weeks, thus allowing for week-to-week fluctuations. Scoring took 5–10 minutes per patient, and this time reduced as therapists became familiar with the tool.

*NPTDA algorithm to calculate estimated therapy hours*: In order to calculate ‘estimated therapy hours/week' from the NPTDA scores, we applied a test algorithm. Within the NPTDA manual, each item scoring level carries an approximate range of hours per week (see Appendix 1b). Our first ‘test algorithm' simply applied the *mid-point* time value for each range (e.g. for a time range 3–4 hours, value 3.5; range 1–2 hours, value 1.5, etc.). Only direct interventions could be compared, as the NPTDA did not record hours for indirect interventions at this point in its development.

*Activity analysis*: Over the same four-week period, each therapist systematically recorded all activity at half-hourly intervals throughout their working week. Activity was coded by each therapist onto a pre-piloted daily timesheet. Activity codes (full list available from the authors on request) were divided into patient-related and non-patient-related activity. Patient-related activity codes were designed to reflect the NPTDA item headings. Patient identity codes were used to assign activities to each individual patient. For simplicity and practical utility in the context of a busy service, where more than one therapeutic activity was undertaken within one 30-minute session, therapists recorded only the principal activity. Completed timesheets were handed at the end of each day and retained by the independent investigator, so that NPTDA estimations at the subsequent ward round were conducted independently of the activity analysis. Out of over 420 timesheets due for the four-week period, only two were missing.

Data were collated for each patient under each item heading in the NPTDA, to build up a series of individual patient records of therapy intervention received over the two-week period. The times were then halved to derive the average hours per week. As well as recording the ‘actual hours' per item for each patient, we also mapped these by reverse transcription to derive NPTDA scores from the activity analysis (‘activity analysis-derived NPTDA' scores), using the time range stated for each scoring level (see Appendix 1b) which, as noted above, varies somewhat for the different items.

#### Data analysis

Data were collated in specifically developed software written in Microsoft Excel, and transferred to SPSS version 11.5 or STATA version 8 for statistical handling.

- The association between ordinal NPTDA scores and actual hours of therapy intervention (derived from activity analysis) was examined using Spearman rank correlations.
- To evaluate the algorithm for converting raw NPTDA scores to therapy hours, the median NPTDA-estimated hours/week were compared with the median ‘actual' hours/week identified from the activity analysis. Comparisons were made for individual items, for each subscale, and for the total. Associations were tested using Spearman rank correlations, and significant differences were tested by paired Wilcoxon signed rank tests.
- We also compared agreement between the ‘activity analysis-derived NPTDA' scores and the ‘team-rated NPTDA' scores in an item-by-item analysis. Agreement was tested using linear-weighted Cohen's kappa statistics (STATA) and interpreted according to Landis and Koch.^19^ Significant differences were tested by paired Wilcoxon signed rank tests.
- The cut-off point for significance was adjusted to *P* < 0.01 to account for multiple tests.

#### Results

There was a moderately strong correlation between total NPTDA ordinal scores and the total intervention hours, as recorded through activity analysis (rho 0.64 *P* < 0.0001).

**Figure 1:**
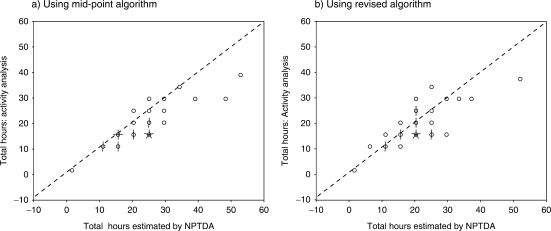
Scattergrams comparing estimations of therapy hours recorded from activity analysis compared with those estimated by the Northwick Park Therapy Dependency Assessment using the two algorithms. In these 'Sunflower' plots, each 'petal' represents a single data pair. The scattergrams demonstrate a reasonably close association. The systematic bias towards overestimation of hours by the mid-point algorithm (a), compared with records of activity analysis is reduced using the revised algorithm (b) (Appendix 2).

Table 1a shows the comparison of the ‘NPTDA-estimated' therapy hours for direct intervention subscales with ‘actual' hours of therapy identified by activity analysis. There was a strong correlation in total hours (rho 0.77, *P* < 0.0001, see Figure 1a), and in all five subscores A–E (rho 0.70–0.93, all *P* < 0.0001). However, the total NPTDA-estimated therapy hours were significantly higher than the ‘actual' hours (median 24 versus 17; Wilcoxon *z* = –3.9, *P* < 0.001) in this analysis. The same trend was observed for all the subscale scores, except for ‘activities of daily living'.

**Figure 2:**
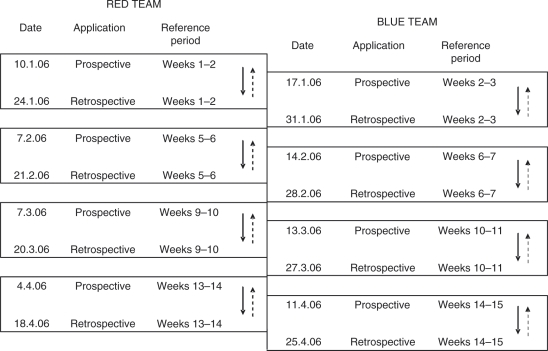
Scoring programme for prospective and retrospective rating. The alternating rating system was employed to avoid excessive rating burden for the team. For any one patient, both prospectively and retrospectively applied scores were collected for the first fortnight of each four-week period, but no scores for the second fortnight.

**Figure 3:**
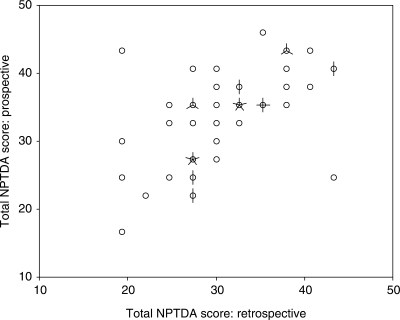
Scattergram of prospective versus retrospective total Northwick Park Therapy Dependency Assessment scores. The scattergram demonstrates a reasonably close association between prospectively-rated ‘intended levels of intervention’ with retrospective ratings of ‘actual intervention’. Where intended levels of intervention were higher than those actually delivered, the differences corresponded to real deviations from intended practice in most instances.

**Table 1:** Comparison NPTDA-estimated hours and actual hours of therapy intervention identified from activity analysis, within each direct intervention subscale

Therapy dependency subscale	Estimated hours/week From NPTDA		Actual hours/week From activity analysis		Comparative analysis			
	Median (IQR)	Range	Median (IQR)	Range	Wilcoxon *Z*-value	*P*-value	Spearman rho	*P*-value
**a) Estimates using the mid-point algorithm**								
Physical subscale	10.0 (7–11.8)	0–18	7.0 (6–9.1)	0–16	−3.4	0.001	0.71	<0.0001
Basic functions	2.0 (0.8–5.3)	0–15	1.5 (0.4–3.8)	0–6.5	−4.2	<0.001	0.93	<0.0001
Activities of daily living	1.5 (0.4–4.8)	0–7.5	2.0 (0.1–3.1)	0–6.0	−1.8	0.07	0.71	<0.0001
Cognitive	4.0 (1.3–6)	0–21.5	1.8 (0.5–3.1)	0–8.3	−3.9	<0.001	0.70	<0.0001
Discharge planning	2.0 (1–7.6)	0–14.5	2.0 (0.4–3.5)	0–10.5	−3.6	<0.001	0.88	<0.0001
Total	24.2 (16.9–27.4)	0–51.5	17.3 (14.6–26.4)	0–40	−3.9	<0.001	0.77	<0.0001
**b) Estimates using the revised algorithm**								
Physical subscale	8.8 (5.6–10.6)	0–18	7.0 (6–9.1)	0–16	−1.7	0.09	0.69	<0.0001
Basic functions	1.3 (0.4–4.3)	0–13.5	1.5 (0.4–3.8)	0–6.5	−2.0	0.05	0.94	<0.001
Activities of daily living	2.3 (2–5)	0–8	2.0 (0.1–3.1)	0–6.0	−2.6	0.011	0.30	0.118
Cognitive	1.0 (0.3–3.8)	0–21	1.7 (0.5–3.1)	0–8.3	−0.2	0.86	0.45	0.014
Discharge planning	2.0 (1–6.1)	0–13.5	2.0 (0.4–3.5)	0–10.5	−3.3	0.001	0.66	<0.0001
Total	20.3 (16–24.9)	0–51.2	17.3 (14.6–26.4)	0–40	−1.4	0.15	0.70	<0.0001

NPTDA, Northwick Park Therapy Dependency Assessment.

Table 2 compares NPTDA scores rated by the team, with scores derived from activity analysis by reverse transcription. Nine of the 22 direct intervention items achieved ‘substantial' or ‘almost perfect' agreement (weighted kappa >0.65), a further eight achieved moderate agreement. Four direct intervention items and two indirect intervention achieved only fair agreement (kappa 0.2–0.4). Amongst these ‘personal self-care', ‘formal family support', and ‘key-working' also showed significant bias towards higher ratings on the team-rated NPTDA scores.

**Table 2:** Agreement between Northwick Park Therapy Dependency Assessment (NPTDA) scores rated by the team and NPTDA scores derived by reverse transcription from activity analysis

Therapy dependency item	Team-rated NPTDA scores		Activity analysis-derived NPTDA scores		Significant differences		Agreement	
	Median (IQR)	Range	Median (IQR)	Range	Wilcoxon *Z*-score	*P*-value	Weighted kappa	Level of agreement
1) Medical management^a^	1 (0–2)	0–4	–	–	–	–	–	–
2a) Splinting/orthotics upper limb	1 (0–2)	0–4	0 (0–2)	0–3	−2.8	0.005	0.75	Substantial
2b) Splinting/orthotics lower limb	0 (0–1)	0–3	0 (0–1)	0–2	−0.7	0.480	0.75	Substantial
3) Seating/wheelchair	2 (1–3)	0–4	1 (0–2)	0–2	−3.1	0.002	0.49	Moderate
4) Physical therapy	4 (3–4)	0–4	3 (2–4)	0–4	−2.1	0.032	0.47	Moderate
5) Trachestomy management	0 (0–0)	0–4	0 (0–0)	0–2	−0.3	0.785	0.65	Substantial
6) Swallowing	0 (0–1)	0–3	0 (0–1)	0–2	−1.6	0.102	0.80	Substantial
7) Nutrition	1 (1–2)	0–4	1 (0–2)	0–4	−2.2	0.029	0.80	Substantial
8) Supported communication	0 (0–2)	0–4	0 (0–1)	0–3	−1.8	0.070	0.48	Moderate
9) Speech and language interventions	2 (1–2)	0–4	1 (0–2)	0–3	−2.4	0.017	0.65	Substantial
10) Personal/self-care	2 (1–3)	0–4	1 (1–2)	0–3	−3.4	0.001	0.41	Fair
11) Domestic/community- based activities	0 (0–1)	0–4	0 (0–1)	0–3	−1.8	0.070	0.69	Substantial
12) Vocational/leisure/ computers/driving	2 (0–2)	0–4	2 (1–2)	0−2	−0.2	0.850	0.57	Moderate
13) Cognitive interventions	0 (0–1)	0–4	0 (0–1)	0–2	−2.0	0.047	0.46	Moderate
14) Behavioural management	0 (0–0)	0–4	0 (0–0)	0–4	−0.4	0.705	0.84	Almost Perfect
15) Emotional/mood	0 (0–2)	0–4	1 (0–2)	0–2	−1.3	0.203	0.55	Moderate
16) Formal family support	0 (0–2)	0–4	0 (0–1)	0–2	−2.6	0.009	0.32	Fair
17) Emotional load on staff^b^	1 (0–2)	0–4	–	–	–	–	–	–
18) Planning discharge/ housing/care package	1 (0–2)	0–4	0 (0–2)	0–4	−2.2	0.029	0.60	Moderate
19) Benefits and finances	1 (0–1)	0–4	0 (0–1)	0–2	−1.2	0.244	0.31	Fair
20) Equipment/adaptation for home	0 (0–1)	0–3	0 (0–0)	0–2	−2.1	0.037	0.43	Moderate
21) Community/home visits	0 (0–0)	0–4	0 (0–0)	0–4	−7.1	0.480	0.81	Almost Perfect
22) Key-working	2 (1–3)	0–4	1 (0–2)	0–3	−3.7	<0.001	0.32	Fair
23) Multidisciplinary meetings	2 (0–2)	0–2	2 (0–2)	0–2	−1.7	0.096	0.35	Fair
24) Reports	0 (0–2)	0–2	0 (0–0)	0–2	−2.1	0.040	0.23	Fair
25) Groups/extra therapies	2 (1–2)	0–2	2 (0–2)	0–2	−2.1	0.038	0.73	Substantial

a Medical staff were excluded from this particular analysis due to staff absence, but they have been included in a subsequent analysis.

b ‘Emotional load on staff’ does not carry a time computation and so could not be compared in this analysis.

#### Adjustment of the algorithm and re-testing

In summary, using the algorithm that applied the mid-point time values, we found a strong overall relationship between the therapy hours identified through activity analysis, and those estimated from the NPTDA, but the latter were consistently higher than those observed.

We therefore explored a number of different algorithms. Simply using the low point of the time range provided a better match overall, but led to underestimation of therapy hours for some items. Our final algorithm was therefore based on a mixture of low and mid-point time values informed by our activity analysis. The algorithm times for each item scoring level are shown in Appendix 2. Figure 1 compares scattergrams of the therapy hours estimated by the two algorithms, and the results of re-analysis using the revised algorithm to compare NPTDA-estimated hours with those derived from activity analysis are summarized in Tables 1b and 3.

**Table 3:** Comparison between ‘NPTDA-estimated’ and ‘actual’ hours of therapy intervention using the revised algorithm

Therapy dependency item	Estimated hours/week From NPTDA		Actual hours/week From activity analysis		Comparative analysis			
	Median (IQR)	Range	Median (IQR)	Range	Wilcoxon *Z*-value	*P*-value	Spearman rho	*P*-value
1) Medical management	0.3 (0–2)	0–6	–	–	–	–	–	–
2a) Splinting/orthotics upper limb	0.3 (0–1)	0–5	0.0 (0–1.5)	0–3	−0.8	0.41	0.77	<0.0001
2b) Splinting/orthotics lower limb	0.0 (0–0.5)	0–3	0.0 (0–0.5)	0–2.5	−0.4	0.71	0.64	<0.0001
3) Seating/wheelchair	1.0 (0–3)	0–5	0.8 (0–2)	0–2.5	−2.2	0.03	0.74	<0.0001
4) Physical therapy	4.0 (3–6.5)	0–11.5	4.5 (3.1–5.5)	0–8	−1.1	0.27	0.58	<0.001
5) Trachestomy management	0.0 (0–0)	0–10	0.0 (0–0)	0–2	0.0	1.00	0.58	<0.001
6) Swallowing	0.0 (0–0.3)	0–3	0.0 (0–0.3)	0–2.5	−0.6	0.56	0.80	<0.0001
7) Nutrition	0.3 (0.1–0.3)	0–7	0.3 (0–0.8)	0–4.5	−0.9	0.35	0.60	<0.0001
8) Supported communication	0.0 (0–1)	0–5	0.0 (0–0.4)	0–3.5	−1.7	0.09	0.60	<0.0001
9) Speech and language interventions	1.1 (0–1)	0–5	0.8 (0–1.5)	0–4	−0.2	0.88	0.73	<0.0001
10) Personal/self-care	2.0 (0.3–4)	0–6	0.8 (0.3–2.0)	0–4.5	−2.4	0.02	0.69	<0.0001
11) Domestic/community- based activities	0.0 (0–0.3)	0–4	0.0 (0–2.5)	0–3	−1.1	0.27	0.70	<0.0001
12) Vocational/leisure/ computers/driving	1.0 (0–1)	0–3	1.0 (0.3–1.8)	0–2.5	−1.9	0.05	0.55	<0.001
13) Cognitive interventions	0.0 (0–0.3)	0–4.5	0.0 (0–0.1)	0–1.5	−1.3	0.20	0.57	<0.001
14) Behavioural management	0.0 (0–0)	0–5	0.0 (0–0)	0–5	−0.1	0.89	0.89	<0.0001
15) Emotional/mood	0.0 (0–1)	0–14	0.3 (0–0.8)	0–1.5	−1.3	0.18	0.65	<0.0001
16) Formal family support	0.0 (0–0.6)	0–6	0.0 (0–0.3)	0–1.5	−2.4	0.02	0.49	0.008
17) Emotional load on staff								
18) Planning discharge/ housing/care package	0.3 (0–1)	0–5	0.0 (0–1.5)	0–4.5	−0.5	0.64	0.69	<0.0001
19) Benefits and finances	0.3 (0–0.3)	0–1	0.0 (0–0.5)	0–1.5	−0.5	0.60	0.42	0.023
20) Equipment/adaptation for home	0.0 (0–0.3)	0–3	0.0 (0–0)	0–1	−1.9	0.05	0.55	<0.001
21) Community/home visits	0.0 (0–0)	0–8	0.0 (0–0)	0–7.5	−0.9	0.34	0.84	<0.0001
22) Key-working	1.0 (0.5–2)	0–5	0.5 (0.3–1.1)	0–3.5	−2.5	0.012	0.45	0.015

NPTDA, Northwick Park Therapy Dependency Assessment.

Using this algorithm, the match appears to be closer. Table 1b demonstrates that there is now no significant difference between the NPTDA-estimated and actual total therapy hours. Although the correlations for each subscale are somewhat less strong than with the mid-point algorithm, the correlation between the total estimates of therapy time remains high (rho 0.70, *P* < 0.0001). Similarly, on item-by-item analysis (Table 3), correlations between estimates of therapy time were significant for all direct intervention items (rho 0.49–0.89, *P* < 0.001), with the exception of two items only (‘benefits and finances' and ‘key-working' – see Discussion).

### Stage 2: Comparison of prospective and retrospectively rated scores

Before the NPTDA can be applied to a hypothetical situation to assess ‘needs' for rehabilitation, it was necessary to determine the extent to which prospective application NPTDA provides a valid advance prediction of the levels of therapy intervention under existing conditions. We compared prospectively rated ‘intended levels of intervention' with retrospective ratings of ‘actual intervention'. We anticipated an approximate relationship but not an exact one, as there are often unpredicted changes in timetabling and staff availability. Moreover, patients' needs can sometimes change, and a flexible rehabilitation team should be able to adjust interventions in response to changing need. Any useful measure of therapy intervention, however, should be able to identify and describe these differences.

#### Design and participants

Stage 2 was undertaken in a second cross-sectional cohort analysis, during the subsequent 15-week period (January–April 2006). We compared the parallel application of prospective and retrospective NPTDA scores. In order to avoid excessive rating burden for the team, instead of applying the NPTDA retrospectively at each fortnightly meeting, prospective and retrospective scoring were alternated as illustrated in Figure 2. Again all patients were included, but only if they were present on the ward for the full two weeks. In total, 51 paired ratings were collected from a total of 31 patients – 16 males and 15 females; mean age 39.2 years (SD 14.6). Twenty-seven had acquired brain injury (14 strokes, 7 traumatic, 6 other, including hypoxia, inflammation and tumour), two had spinal cord injury and two Guillain–Barré syndrome.

#### Data collection

At the beginning of each two-week block, the treating team rated ‘prospective NPTDA' scores for each patient, based on the average level of input per week they *intended* to give for each item during that period. Scores were rated during the routine goal-planning meeting, during which the team normally sets short-term goals and plans treatment for the coming fortnight. At the end of the same period, ‘retrospective NPTDA' scores were assigned by the team based on the average level of interventions *actually given* over that same two-week period. NPTDA scores were retained by the investigator rather than being filed in the patient records, so that at each scoring point, therapists were unable to refer to any previous scores, and in this sense were ‘blinded' to the scores they had given two weeks earlier.

#### Data analysis

As for stage 1, associations between prospective and retrospective scores or hours were tested using Spearman rank correlations. Agreement was tested using linear-weighted Cohen's kappa statistics. Significant differences were tested by paired Wilcoxon signed rank tests. The cut-off point for significance was again adjusted to *P* < 0.01 to account for multiple tests. In the absence of pre-existing data to make a formal power calculation, our sample size was based on the crude calculation of 2*K*^2^ which, for a 5-point scale, is 50.

#### Results

Table 4 summarizes the comparison between prospective and retrospectively rated scores. There was a strong association between the total NPTDA scores (rho 0.61, *P* < 0.0001, see Figure 3) and subscale scores were also significantly correlated (rho 0.44–0.81, all *P* < 0.001). On item-by-item analysis, weighted kappas ranged from 0.28 to 0.77, with 12/22 direct intervention items showing ‘moderate' to ‘substantial' agreement (kappa >0.40), but 10 showed only ‘fair' agreement. Agreement for the subscale scores was ‘fair' to ‘moderate.'

**Table 4:** Summary of comparison for prospective and retrospective Northwick Park Therapy Dependency Assessment scores

Therapy dependency item	Prospective scores		Retrospective scores		Comparison					
	Median (IQR)	Range	Median (IQR)	Range	Wilcoxon *Z*-value	*P*-value	Spearman rho	*P*-value	Weighted kappa	Level of agreement
1) Medical management	2 (2–3)	0–4	2 (1–3)	0–4	−1.61	0.11	0.50	<0.0001	0.40	Fair
2a) Splinting/orthotics upper limb	1 (0–3)	0–4	0 (0–2)	0–4	−1.27	0.21	0.79	<0.0001	0.63	Substantial
2b) Splinting/orthotics lower limb	1 (0–2)	0–3	1 (0–2)	0–3	−0.47	0.64	0.45	<0.0001	0.40	Fair
3) Seating/wheelchair	2 (1–2)	0–4	2 (1–2)	0–4	−1.18	0.24	0.63	<0.0001	0.48	Moderate
4) Physical therapy	3.5 (3–4)	0–4	3.5 (3–4)	0–4	−0.25	0.81	0.24	0.08	0.28	Fair
5) Trachestomy management	0 (0–0)	0–0	0 (0–0)	0–0	0.00	1.00	N/A^a^	N/A	N/A	N/A
6) Swallowing	0 (0–0)	0–3	0 (0–0)	0–4	−1.07	0.29	0.75	<0.0001	0.64	Substantial
7) Nutrition	1 (0–2)	0–4	1 (0–2)	0–4	−2.23	0.03	0.47	<0.0001	0.43	Moderate
8) Supported communication	0 (0–0)	0–4	0 (0–0)	0–2	−0.81	0.42	0.73	<0.0001	0.48	Moderate
9) Speech and language interventions	2 (0–3)	0–4	2 (0–3)	0–4	−0.91	0.37	0.90	<0.0001	0.77	Substantial
10) Personal/self-care	2 (2–2)	0–4	2 (1–2)	0–4	−1.18	0.24	0.49	<0.0001	0.35	Fair
11) Domestic/community- based activities	2 (0–2)	0–4	1 (0–2)	0–4	−1.62	0.10	0.29	0.04	0.29	Fair
12) Vocational/leisure/ computers/driving	2 (0–2)	0–3	2 (0–2)	0–4	−0.69	0.49	0.37	<0.0001	0.34	Fair
13) Cognitive interventions	0 (0–2)	0–4	0 (0–1)	0–4	−1.19	0.23	0.45	<0.0001	0.47	Moderate
14) Behavioural management	0 (0–0)	0–4	0 (0–0)	0–4	−1.29	0.20	0.67	<0.0001	0.61	Substantial
15) Emotional/mood	1 (0–2)	0–4	2 (0–2)	0–4	−0.84	0.40	0.67	<0.0001	0.66	Substantial
16) Formal family support	0 (0–1)	0–4	0 (0–1)	0–4	−0.31	0.76	0.46	<0.0001	0.39	Fair
17) Emotional load on staff	0 (0–2)	0–4	0 (0–2)	0–4	−0.46	0.65	0.66	<0.0001	0.52	Moderate
18) Planning discharge/housing/ care package	2 (1–3)	0–4	1 (0–3)	0–4	−0.63	0.53	0.46	<0.0001	0.41	Moderate
19) Benefits and finances	1 (1–2)	0–3	1 (1–2)	0–4	−0.13	0.90	0.55	<0.0001	0.50	Moderate
20) Equipment/adaptation for home	1 (0–2)	0–4	0 (0–2)	0–3	−2.95	0.003	0.42	<0.0001	0.31	Fair
21) Community/home visits	0 (0–3)	0–4	0 (0–2)	0–4	−1.71	0.09	0.38	0.006	0.38	Fair
22) Key-working	2 (1–2)	0–4	2 (1–2)	1–3	−1.19	0.24	0.35	0.012	0.31	Fair
23) Multidisciplinary meetings	2 (0–2)	0–2	2 (0–2)	0–2	0.00	1.00	0.77	<0.0001	0.77	Substantial
24) Reports	1 (0–1)	0–2	0 (0–2)	0–2	−2.51	0.012	0.59	<0.0001	0.50	Moderate
25) Groups/extra therapies	2 (2–2)	0–2	2 (2–2)	0–2	−0.56	0.57	0.35	0.012	0.30	Fair
26) Clinics	0 (0–2)	0–2	0 (0–2)	0–2	−0.63	0.53	0.60	<0.0001	0.60	Moderate
Subscale scores										
Physical subscale	9.5 (8–11)	3–15	9.5 (7–11.5)	3–16	−1.76	0.08	0.71	<0.0001	0.52	Moderate
Basic functions	4 (1–5.5)	0–9	3 (2–5)	0–8	−1.57	0.12	0.81	<0.0001	0.54	Moderate
ADL	5 (3–6)	1–9	4 (2–6)	1–10	−2.01	0.05	0.44	<0.001	0.29	Fair
Cognitive	2 (1–6)	0–16	3 (1–6)	0–13	−0.57	0.57	0.64	<0.0001	0.55	Moderate
Discharge planning	7 (5–9.5)	1–15	6 (4–8)	1–13	−2.53	0.011	0.53	<0.0001	0.31	Fair
Indirect interventions	4 (3.5–6)	2–8	4 (3–6)	0–8	−0.74	0.46	0.55	<0.0001	0.44	Moderate
Total NPTDA	35 (28–38.5)	16–45	30 (26.6–35.5)	18–43	−3.30	<0.001	0.61	<0.0001	0.38	Fair

a In this particular series, unlike the earlier one, no patient had a tracheostomy.

Overall there was a small tendency to overestimate predicted input through prospective scoring, leading to a significant difference in total scores (Wilcoxon *z* = –3.30, *P* < 0.001). There was some considerable variation, however. Within the individual items, only scores for ‘equipment provision' were significantly different.

## Discussion and conclusions

In the absence of an established gold standard against which to test criterion validity of the NPTDA, we used activity analysis to examine concurrent validity in stage 1. We found a strong overall relationship between the two estimates of therapy hours. However, those derived from the NPTDA using the mid-point algorithm were consistently higher than those observed through activity analysis, and there were several possible explanations for this bias:

1. Activity recording may have been incomplete,
2. The NPTDA may have overestimated therapy intervention, either because the rating therapists over-estimated scores or this ‘mid-point' algorithm overestimated the time taken.

Strenuous efforts had been made to ensure complete recording of activity analysis, with only two forms missing over the study period. The decision to record only the dominant activity for each session may have led to some inaccuracies, but should have equally under- and overestimated time for different activities, so avoiding systematic bias. However, short activities such as phone calls may not have been adequately captured, which might explain the poor correlation in items such as key-working and discharge planning.

Review discussions with the team revealed the following:

1. Overestimation by scoring therapists was problematic in certain areas – especially those where intervention is mainly undertaken by assistant staff who are not present during scoring in the main ward round (interventions for ‘personal self-care' and ‘benefits and finances' were examples of this).
2. Overall, however, the team agreed that the mid-point algorithm overestimated times and required readjustment.
3. There were large variations in therapy hours where a score of 4 was applied, as some interdisciplinary interventions involved only short periods. A ‘3.5 level' was therefore introduced to identify short interdisciplinary interventions, and to avoid overestimation of therapy needs in these circumstances.

In stage 2, there was a strong relationship between prospectively allocated and retrospective scores, but prospective scores were fairly consistently higher. Again there may be several reasons:

1. Staff may be over-optimistic about their ability to fit all their duties into the time available
2. Planned sessions may be cancelled because of patient illness, refusal or unavailability; staff sickness or unexpected leave; or other crisis intervention.
3. Staff may fail to remember all their interventions, and so rate lower scores retrospectively.

Team debriefing identified a number of issues that were thought to have contributed to the discrepancy:

1. The study period coincided with a new UK-wide policy, requiring staff to re-negotiate their working contracts. Staff frequently complained that the extra administration involved impacted on the clinical care of patients during this period.
2. Other departures from expected plans included:

- unexpected absence of one staff member on long-term compassionate leave,
- some documented episodes of intercurrent illness (for both patients and staff)
- failure of delivery of specialist equipment items, requiring discharge planning arrangements to be altered.

After reviewing the scores for specific instances of disagreement, the team agreed that, in the majority of cases, discrepancies between NPTDA scores had appropriately identified a real deviation from the level of intervention intended. This provides some support for the notion that the tool may have potential future application in describing the difference between the level of input provided, and hypothetical situations such as level of service ‘intended' (as tested here) or the level of services ‘needed' – although this must be tested separately. In the meantime, it underlines the importance of specifying the mode of application when results are reported.

In comparison with the NPDS, on which the NPTDA is modelled, it is important to recognize that the estimation of ‘requirements for therapy' is inevitably more subjective than that for ‘basic care needs' which most people would reasonably regard as essential. Previous experience suggests that these tools continue to evolve and develop over a decade or more, and much wider testing and validation will required before the NPTDA can be accepted on a similar footing to the NPDS as an estimation of ‘needs'. That said, the potential for hypothetical application makes the NPTDA unique, in comparison with other existing tools^5,7–12^ which can only be applied to describe interventions that were actually given. However, these other tools offer the advantage of more detailed analysis of specific therapy interventions than is possible with the broad-brush approach of the NPTDA. In this respect, the two different approaches may be found to complement each other and may usefully be applied in combination in future attempts to characterize black box of rehabilitation.^20^

There are a number of clear limitations to this study:

1. This first validation study forms only one part of the on-going evaluation of the tool; other aspects such as reliability, responsiveness, utility, etc. are currently being addressed and will be presented for publication separately.
2. There were a number of methodological challenges:

- Rating bias: we recognize a potential for bias as the same therapists had to record their NPTDA scores and the activity analysis (in stage 1) and both prospective and retrospective ratings (in stage 2). In order to reduce bias, ratings were handed in to the investigator as soon as they were completed, and so were not available to staff when subsequent ratings were made. In addition, the data volumes were large – during a two-week period each full-time therapist would record some 160 items of activity analysis across their caseload – making it unlikely that they would carry these in their memory whilst rating the NPTDA scores. Nevertheless some potential for rating bias must inevitably exist.
- Incomplete capture of activities: The decision to record only the dominant activity in each 30-minute period may have underestimated time spent on short tasks during the activity analysis. From the information perspective, shorter sample periods (e.g. every 10–15 minutes) are ideal, but are even more burdensome to collect in the course of routine practice, and may well have been more inaccurate in the end due to clinician burn-out.
- Sample size: Activity analysis is time-consuming both for clinicians and for therapists. Whilst our analysis captured activity for approximately 420 therapist-days over the study period (20 working days for 20.3 WTE) generating a large quantity of data, this was in reality a small sample involving just 17 different patients on a single unit, and caution must be applied in generalizing these findings to other services.

The NPTDA has been developed in the context of post-acute inpatient neurological rehabilitation. This particular service was chosen for its cohort of patients with complex rehabilitation needs, but further work is now required to test the algorithm in different settings, with different teams and different patient groups and other areas of rehabilitation. We have started to explore its adaptation for use with children and cognitive behavioural settings.

Despite the recognized limitations, this article describes the initial development of a potentially important tool to inform clinical practice. The results presented provide encouraging early support for its potential to provide a reasonable estimate of therapy interventions, which is practical to apply in the context of routine clinical care. Further exploration and evaluation is now warranted.

**Clinical messages**

- The Northwick Park Therapy Dependency Assessment is a tool to measure therapy needs and interventions in neurorehabilitation and to quantify these in terms of staff time.
- In this first evaluation study it provided a reasonable estimate of therapy hours.
- Further development and evaluation are now underway.

Full details of the NPTDA and computerized software are available from the corresponding author.

## Appendix

**Appendix 1a:** Domains of the Northwick Park Therapy Dependency Assessment (NPTDA)

Domain	Items	Lead discipline	Scores	Totals
A: Physical handing programme	1) Medical management	Doctor	0–4	
	2a) Splinting orthotics (upper limb)	O/T	0–4	
	2b) Splinting orthotics (lower limb)	Physio	0–4	
	3) Seating/wheelchair	O/T	0–4	
	4) Physical therapy–active/passive handling	Physio	0–4	0–20
B: Basic functions	5) Tracheostomy management	SLT	0–4	
	6) Swallowing	SLT	0–4	
	7) Nutrition	Dietitian	0–4	
	8) Supported communication	SLT	0–4	
	9) Speech and language interventions	SLT	0–4	0–20
C: Activities of daily living	10) Personal/self-care	O/T	0–4	
	11) Domestic/community-based activities	O/T	0–4	
	12) Vocational/leisure/computers/driving	O/T	0–4	0–12
D: Cognitive/psychosocial/ family support	13) Cognitive interventions	Psychology	0–4	
	14) Behavioural management	Psychology	0–4	
	15) Emotional/mood	Psychology	0–4	
	16) Formal family support	Psychology	0–4	
	17) Emotional load on staff	Psychology	0–4	0–20
E: Preparing for discharge	18) Planning discharge/housing/care package	SW	0–4	
	19) Benefits and finances	SW	0–4	
	20) Equipment/adaptations for home	O/T	0–4	
	21) Community/home visits	O/T	0–4	
	22) Key-working	…	0–4	0–20
F: Indirect interventions + additional activities	23) Multidisciplinary meetings	State	0–2	
	24) Reports	State	0–2	
	25) Groups/extra therapies	State	0–2	
	26) (Accompanied) clinic attendance	State	0–2	0–8
G: Special input	27) Special facilities	(Select from lists)	Text	
	28) Special equipment hire	(Select from lists)	Text	
	29) Investigations	(Select from lists)	Text	
	30) Procedures	(Select from lists)	Text	
			Total	0–100

O/T, occupational therapist; SLT, speech and language therapist; SW, social worker.

**Appendix 1b:** Example of the general scale structure for most items

Score	Approximate hours/week^a^ (Example only)	Descriptor	Level description of therapy input
	Range		
0	0	None	Not relevant, or no planned therapy at the current time
1	<1	Low	Minimal intervention, or review only
2	1–2	Medium	Basic intervention, or intervention by assistant only
3	3–4	High	More intensive intervention by qualified therapist +/− assistant
3.5^b^	<4	Interdisciplinary	Interdisciplinary intervention, but for limited total time
4	>4	Complex	Interdisciplinary intervention, and/or very high intensity input

a The ‘approximate hours/week’ varies for the different items. Therapists are encouraged to rely primarily on the level descriptions for scoring, but the time range is designed to provide a rough guide to assist scoring. Times were derived in development through observational analysis.

b The initial version used in our first validation study did not include a 3.5 score. This was added subsequently to avoid overestimation of therapy needs requiring simultaneous intervention from several disciplines, but only for a short period.

**Appendix 2:** Revised algorithm for NPTDA estimation of hours/week in relation to scores

Items	Lead discipline	Hours per week ascribed to lead discipline				Individually recorded hours per discipline	
		0	1	2	3	3.5	4	
1) Medical management	Doctor	0	1	2.5	4.5	(–)	6	
2a) Splinting orthotics (upper limb)	O/T	0	0.5	1	3	Individualized	
2b) Splinting orthotics (lower limb)	Physio	0	0.5	1	3	Individualized	
3) Seating/wheelchair	O/T	0	0.5	1	3	Individualized	
4) Physical therapy – active/passive handling	Physio	0	1	2.5	4.5	Individualized	
5) Tracheostomy management	SLT	0	0.5	1	3	Individualized	
6) Swallowing	SLT	0	0.5	1	3	Individualized	
7) Nutrition	Dietitian	0	0.25	0.75	2	Individualized	
8) Supported communication	SLT	0	0.5	1	3	Individualized	
9) Speech and language interventions	SLT	0	0.5	1	3	Individualized	
10) Personal/self-care	O/T	0	0.5	1	3	Individualized	
11) Domestic/community-based activities	O/T	0	0.5	1	3	Individualized	
12) Vocational/leisure/computers/driving	O/T	0	0.5	1	3	Individualized	
13) Cognitive interventions	Psychology	0	0.5	1	3	Individualized	
14) Behavioural management	Psychology	0	0.5	1	3	Individualized	
15) Emotional/mood	Psychology	0	0.5	1	3	Individualized	
16) Formal family support	Psychology	0	0.5	1	3	Individualized	
17) Emotional load on staff	Psychology	–	–	–	–	Individualized	
18) Planning discharge/housing/care package	SW	0	0.5	1	3	Individualized	
19) Benefits and finances	SW	0	0.5	1	3	Individualized	
20) Equipment/adaptations for home	O/T	0	0.5	1	3	Individualized	
21) Community/home visits	O/T	0	1	3	5	Individualized	
22) Key-working	…	0	0.5	1	3	Individualized	
23) Multidisciplinary meetings	State	0	Individualized	(–)	(–)	(–)	
24) Reports	State	0	Individualized	(–)	(–)	(–)	
25) Groups/extra therapies	State	0	Individualized	(–)	(–)	(–)	
26) (Accompanied) clinic attendance	State	0	Individualized	(–)	(–)	(–)	

O/T, occupational therapist; SLT, speech and language therapist; SW, social worker.

The algorithm is designed to provide a generic estimate of the implications for staff time associated with each item level of the NPTDA. The times were derived from the initial activity analysis presented in this article, but require testing and further development for other settings.

The computerized NPTDA data entry sheet automatically ascribes the hours of intervention shown for each level to the lead discipline as shown for levels 0–3. When levels 3.5 or 4 are entered staff are prompted to supply the estimated times for each discipline involved.

## References

[1] Kwakkel G Impact of intensity of practice after stroke: issues for consideration Disabil Rehabil 2006; 28:823–30 16777769 10.1080/09638280500534861

[2] Shiel A Burn JPS Henry D The effects of increased rehabilitation therapy after brain injury: results of a prospective controlled trial Clin Rehabil 2001; 15:501–14 11594640 10.1191/026921501680425225

[3] Slade A Chamberlain MA Tennant A A randomised controlled trial to determine the effect of intensity of therapy on length of stay in a neurological rehabilitation setting J Rehabil Med 2002; 34:260–66 12440799 10.1080/165019702760390347

[4] Gladman J Barer D Langhorne P Specialist rehabilitation after stroke (editorial) BMJ 1996; 312:1623–24 8664701 10.1136/bmj.312.7047.1623 PMC2351345

[5] Ballinger C Ashburn A Low J Roderick P Unpacking the black box of therapy–a pilot study to describe occupational therapy and physiotherapy interventions for people with stroke Clin Rehabil 1999; 13:301–309 10460118 10.1191/026921599673198490

[6] De Jong G Horn SD Gassaway JA Slavin MD Dijkers MP Toward a taxonomy of rehabilitation interventions: Using an inductive approach to examine the ‘black box'of rehabilitation Arch Phys Med Rehabil 2004; 85:678–86 15083447 10.1016/j.apmr.2003.06.033

[7] De Wit L Kamsteegt H Yadav B Verheyden G Feys H De Weerdt W Defining the content of individual physiotherapy and occupational therapy sessions for stroke patients in an inpatient rehabilitation setting. Development, validation and inter-rater reliability of a scoring list Clin Rehabil 2007; 21:450–59 17613566 10.1177/0269215507074385

[8] Bode RK Heinemann AW Semik P Mallinson T Patterns of therapy activities across length of stay and impairment levels: peering inside the ‘black box' of inpatient stroke rehabilitation Arch Phys Med Rehabil 2004; 85:1901–908 15605324 10.1016/j.apmr.2004.02.023

[9] Latham NK Jette DU Slavin M Physical therapy during stroke rehabilitation for people with different walking abilities Arch Phys Med Rehabil 2005; 86 suppl 2 S41–S50 16373139 10.1016/j.apmr.2005.08.128

[10] Richards LG Latham NK Jette DU Rosenberg L Smout RJ DeJong G Characterizing occupational therapy practice in stroke rehabilitation Arch Phys Med Rehabil 2005; 86 suppl 2 S51–S60 16373140 10.1016/j.apmr.2005.08.127

[11] van Langeveld SA Post MW van Asbeck FW Postma K Leenders J Pons K Feasibility of a classification system for physical therapy, occupational therapy, and sports therapy interventions for mobility and self-care in spinal cord injury rehabilitation Arch Phys Med Rehabil 2008; 89:1454–59 18674980 10.1016/j.apmr.2007.12.044

[12] van Langeveld SA Post MW van Asbeck FW Postma K Ten Dam D Pons K Development of a classification of physical, occupational, and sports therapy interventions to document mobility and self-care in spinal cord injury rehabilitation J Neurol Phys Ther 2008; 32:2–7 18463549 10.1097/NPT.0b013e3181663533

[13] Turner-Stokes L Tonge P Nyein K Hunter M Nielson S Robinson The Northwick Park Dependency Score (NPDS): a measure of nursing dependency in rehabilitation Clin Rehabil 1998; 12:304–18 9744666 10.1191/026921598669173600

[14] Post MW Visser-Meily JM Gispen LS Measuring nursing needs of stroke patients in clinical rehabilitation: a comparison of validity and sensitivity to change between the Northwick Park Dependency Score and the Barthel Index Clin Rehabil 2002; 16:182–89 11926176 10.1191/0269215502cr474oa

[15] Hatfield A Hunt S Wade DT The Northwick Park Dependency Score and its relationship to nursing hours in neurological rehabilitation J Rehabil Med 2003; 35:116–20 12809193 10.1080/16501970310010457

[16] Skinner A Turner-Stokes L The use of standardised outcome measures for rehabilitation in the UK Clin Rehabil 2005; 20:609–15 16894804 10.1191/0269215506cr981oa

[17] Svensson S Sonn U Sunnerhagen KS Reliability and validity of the Northwick Park Dependency Score (NPDS) Swedish version 6.0 Clin Rehabil 2005; 19:419–25 15929511 10.1191/0269215505cr808oa

[18] Turner-Stokes L The Northwick Park Therapy Dependency Score (NPTDA): Development, preliminary evaluation and application. Department of Health R&D Project Grant Report ref. 030/0066; 2006

[19] Landis JR Koch GG The measurement of observer agreement for categorical data Biometrics 1977; 33:159–74 843571

[20] Khan F Pallant JF Zhang N Turner-Stokes L Clinical practice improvement approach in multiple sclerosis rehabilitation: a pilot study. Submitted 2009 10.1097/MRR.0b013e328338b05f 20410826

